# One-year outcomes of conventional and accelerated collagen crosslinking in progressive keratoconus

**DOI:** 10.1038/srep14425

**Published:** 2015-09-25

**Authors:** Vanissa W. S. Chow, Tommy C. Y. Chan, Marco Yu, Victoria W. Y. Wong, Vishal Jhanji

**Affiliations:** 1 Hong Kong Eye Hospital, Hong Kong SAR, China; 2 Department of Mathematics and Statistics, Hang Seng Management College, Hong Kong; 3 Department of Ophthalmology & Visual Sciences, The Chinese University of Hong Kong, Hong Kong SAR, China

## Abstract

We compared one-year outcomes of conventional (3 mW/cm^2^, 365-nm ultraviolet-A light, 30 minutes) and accelerated (18 mW/cm^2^, 365-nm ultraviolet-A light, 5 minutes) collagen crosslinking (CXL) in patients with progressive keratoconus. Main outcome measures were change in keratometry, uncorrected visual acuity (UCVA), and best-corrected visual acuity (BCVA). Nineteen patients in each group completed 1-year follow-up. Preoperatively, there were no inter-group differences for age, keratometry, corneal thickness, and spherical equivalent (p > 0.127). One year postoperatively, maximum and minimum keratometry were flattened by 1.6 diopters (p < 0.023) and 2 diopters (p < 0.047) respectively after conventional CXL, and, 0.47 diopters (p = 0.471) and 0.19 diopters (p = 0.120) respectively after accelerated CXL. Association analysis showed significant negative association between baseline maximum keratometry and change in maximum keratometry after accelerated CXL (p = 0.002) but not after conventional CXL (p = 0.110). Corneal thickness was reduced significantly in both groups (p = 0.017). An improvement in UCVA (p < 0.001) and BCVA (p < 0.022) was noted in both groups along with a reduction in spherical equivalent postoperatively (p < 0.026). There were no inter-group differences for any of the parameters postoperatively (p > 0.184). Although no statistically significant differences were observed between both treatment modalities, a more effective topographic flattening was observed with conventional CXL as compared to accelerated CXL in this study.

Keratoconus is a bilateral, non-inflammatory corneal ectasia characterized by progressive corneal thinning and protrusion leading to progressive myopia, irregular astigmatism and corneal scarring^1^. Corneal collagen crosslinking (CXL) has been shown to stop disease progression in keratoconus^2^. This procedure, utilizing riboflavin and ultraviolet-A light, significantly increases the biomechanical strength of the cornea by photochemical crosslinking of individual collagen fibers^3^. There is ample evidence that conventional CXL following the Dresden protocol of 3 mW/cm^2^ irradiance for 30 minutes retards the progression of keratoconus with favorable clinical and topographic results^2^,^4^,^5^,^6^,^7^,^8^.

Accelerated CXL has been developed to shorten the duration of the procedure by increasing illumination intensity. Protocols are generally developed based on the Bunsen-Roscoe law of reciprocity, keeping a constant radiant exposure of 5.4 J/cm^2^. While clinical studies of various settings of 5.4 J/cm^2^ demonstrate promising efficacy^9^,^10^,^11^,^12^,^13^,^14^,^15^, *ex-vivo* studies on corneal stiffness have produced conflicting results^16^,^17^. Recently, comparative studies between conventional CXL and 30 mW/cm^2^ accelerated CXL seem to show that both treatment protocols are equally safe and effective^14^,^15^. However, these studies either used different preparations of riboflavin or different machines for irradiation between the protocols, which may confound the outcome of the studies.

The purpose of the current study was to compare the clinical and topographic effects between conventional CXL (3 mW/cm^2^ for 30 minutes) and accelerated CXL (18 mW/cm^2^ for 5 minutes) in progressive keratoconus. In this study, the treatment protocols were designed to provide equivalent radiant exposures using the same formulation of riboflavin to address potential confounding variables in prior comparative studies of ACXL and CCXL.

## Results

Thirty-eight eyes (19 eyes in each group) of 32 patients were included in the final analysis. Two eyes of two patients were excluded from the final analysis due to an incomplete follow-up. The mean age of patients in conventional CXL and accelerated CXL group was 27.8 ± 10.9 years and 26.3 ± 3.7 years respectively (p = 0.677). There were 13 males in conventional CXL group and 12 males in accelerated CXL group (p = 1.000). The mean progression in maximum keratometry 1 year before the surgery was 1.60 ± 0.27 diopters in conventional CXL group and 1.79 ± 0.30 diopters in accelerated CXL group (p = 0.635). The mean progression in astigmatism 1 year before the surgery was 1.04 ± 0.14 diopters in conventional CXL group and 1.22 ± 0.14 in accelerated CXL group (p = 0.373).

Table 1 shows the baseline parameters in both groups. There was no statistically significant difference between maximum keratometry, minimum keratometry, thinnest corneal thickness, central corneal thickness, spherical equivalent and endothelial cell density in both groups (p > 0.127). Baseline UCVA (p = 0.038) and BCVA (p = 0.002) were slightly better in the accelerated CXL group.

Postoperatively, maximum keratometry flattened by 1.6 diopters (p < 0.023) and minimum keratometry flattened by 2 diopters in the conventional CXL group (p < 0.047). The corresponding values in accelerated CXL group were 0.47 diopters (p = 0.471) and 0.19 diopters (p = 0.120). However, there were no inter-group differences in the changes in keratometry values between conventional CXL and accelerated CXL at 1 year postoperatively (Table 2). The corneal thicknesses (thinnest corneal thickness and central corneal thickness) decreased significantly postoperatively in both groups (p > 0.017), although no inter-group difference was found. Central corneal thickness reduced by 23.56 μm in conventional CXL group and 22.63 μm in accelerated CXL group (p = 0.606). The mean change in thinnest corneal thickness was 29.91 μm and 22.38 μm in conventional CXL and accelerated CXL group respectively at the end of 1 year (p = 0.305) (Table 2).

After 1 year of treatment, both treatment groups had a significant improvement in UCVA (accelerated CXL, p < 0.001; conventional CXL, p < 0.001) and BCVA (accelerated CXL, p < 0.021; conventional CXL, p < 0.022). The magnitude of improvement was similar in both groups without any statistical significance (p > 0.430). Spherical equivalent also decreased significantly in both groups (p < 0.026), with no inter-group difference (p = 0.554) (Table 2).

There was a significant decrease in endothelial cell density after accelerated CXL (p = 0.009) but not after conventional CXL (p = 0.190) (Table 1). However, the mean loss of cells was clinically inconsequential and comparable between both groups (p = 0.817) (Table 2).

Two cases in accelerated CXL group (2/19, 10.5%) and 3 cases in conventional CXL group (3/19; 15.8%) developed mild, visually insignificant corneal haze (p = 1.000). The corneal haze resolved within 3 months postoperatively in all cases.

Association analysis was performed between the baseline maximum keratometry, BCVA and UCVA and the change in maximum keratometry, BCVA and UCVA, respectively, based on linear mixed effect models. A significant negative association was found between baseline maximum keratometry and change in maximum keratometry in accelerated CXL group, suggesting higher preoperative maximum keratometry values were associated with greater reduction in maximum keratometry values. A significant negative association was also found between UCVA and UCVA in both groups (Table 3).

## Discussion

In this study, the corneal flattening effect of conventional CXL was statistically significant as compared to baseline unlike the corneal flattening of accelerated CXL, at the end of one year postoperatively. Except for central corneal thickness and thinnest corneal thickness, clinical and topographic parameters were stable in both treatment groups. This is in accordance with the results published in the literature^2^,^4^,^5^,^6^,^7^,^8^,^9^,^10^,^11^,^12^,^13^,^14^,^15^,^18^. We observed a significant improvement in UCVA, BCVA and spherical equivalent in both groups. Previous studies have demonstrated similar functional improvement after CXL^2^,^5^,^6^,^7^,^9^,^15^,^18^. This has been attributed to an improved regularity of the corneal shape after CXL.

In terms of topographic parameters, though apparently not clinically significant, we noticed a greater reduction in keratometry in the conventional CXL group than in the accelerated CXL group. Maximum and minimum keratometry were significantly flattened one year after conventional CXL, while those after accelerated CXL remained stable. This may be partly explained by the small though insignificant difference in preoperative maximum keratometry between the two groups (54.93D in conventional CXL group; 51.96D in accelerated CXL group; p = 0.235), as previous studies have shown that greater topographic flattening may occur with initially steeper corneas^19^,^20^,^21^,^22^,^23^. Association analysis in our study showed that greater maximum keratometry values were associated with a stronger flattening effect after accelerated CXL. Interestingly, no such association was observed in cases undergoing conventional CXL. On the other hand, worse baseline UCVA was associated with greater change in postoperative UCVA in both accelerated and conventional CXL groups. A recently published comparative study between four protocols of CXL in eyes with preoperative keratometry comparable to our study (steep keratometry of 48.6 to 50.5D) showed that conventional CXL of 3 mW/cm^2^ had greater flattening effect compared to accelerated CXL protocols of 9 mW/cm^2^, 18 mW/cm^2^ and 30 mW/cm^2^ at one year^18^. We have also noted, in another study with a different patient cohort, that 18 mW/cm^2^ accelerated CXL treatment was not able to induce corneal flattening at one year in eyes with baseline maximum keratometry <58D, with mean change of 1.00 ± 1.63 D in maximum keratometry at one year^23^. The authors attributed this to the potentially reduced biomechanical effect of accelerated CXL treatments^18^,^23^. Wernli *et al.* observed that an equivalent stiffness increase could be achieved up to an illumination intensity of approximately 40 to 45 mW/cm^2^, corresponding to illumination time of approximately 2 minutes. For higher intensities ranging from 50 mW/cm^2^ up to 90 mW/cm^2^, no statistically significant stiffness increase could be achieved therefore citing the non-applicability of the Bunsen-Roscoe reciprocity law for short illumination time and high intensities^16^. Hammer *et al.* observed a decreasing trend in Young’s modulus with increasing irradiances, reaching no statistically significant difference between the 18 mW/cm^2^ and the control group. The authors proposed that intrastromal oxygen diffusion capacity and increased oxygen consumption associated with higher irradiances may be a limiting factor leading to reduced treatment efficiency^17^. A shallower demarcation line was also observed in accelerated CXL^14^,^24^, suggesting a reduced treatment effect compared to conventional CXL. The authors also found that a 40% increase in irradiation time was required during accelerated CXL to achieve a similar depth in demarcation line as conventional CXL^25^.

Previous studies did not find any difference in keratometry in their clinical studies comparing conventional CXL and accelerated CXL^14^,^16^. Tomita *et al.* used different machines as well as different preparations of riboflavin in their conventional and accelerated CXL protocols^14^. Although Hashemian *et al.* did not mention the machine type used for accelerated CXL treatment in their study, the authors used high-energy (30 mW/cm^2^ for 3 minutes) irradiation for accelerated CXL. Machines may differ in their top-hat profiles and eye-tracing ability, rendering their total dose intensity different from their intended energy of 5.4 J/cm^2^. In the current study, the treatment protocols were designed to provide equivalent radiant exposures using the same formulation of riboflavin to address potential confounding variables in prior comparative studies of accelerated and conventional CXL. Although CXL was performed using machines from different manufacturers in our study, the settings for both devices were similar in terms of wavelength of ultraviolet light, working distance, illumination diameter, and, light emission. All other perioperative parameters as well as postoperative treatment were identical in both groups.

Tomita *et al.* used 0.1% riboflavin with hydroxypropyl methylcellulose for 15 minutes, instead of 0.1% riboflavin with 20% dextran for 30 minutes in their accelerated CXL protocol. Intraoperative corneal thinning has been observed using standard riboflavin with 20% dextran^26^,^27^. Different riboflavin compositions have been associated with variations in the central corneal thickness^28^, where riboflavin solution without dextran could lead to an increase in the corneal thickness during CXL^29^. Wollensak also showed that the riboflavin film was important in achieving correct corneal UVA irradiance^30^. Whether different preparations of riboflavin have any effect on the clinical efficacy of the procedure remains to be elucidated.

There was a statistically significant reduction in thinnest corneal thickness and central corneal thickness one year after both treatment protocols in the current study. The clinical significance of such change is not fully understood. Though it may represent an underlying progression of the disease, it may well be due to an increased compactness of the crosslinked cornea, as suggested by Greenstein *et al.*^31^ In a randomized controlled study by Witting-Silva *et al.*, a significant corneal thinning of 33 μm was observed despite clinical and topographic improvement 3 years after CXL, while the control group only had 10 μm of corneal thinning^2^. The decreased corneal thickness after collagen crosslinking may also be attributed to the use of Orbscan. The corneal thickness measurements with Orbscan have been shown to be affected by the presence of corneal haze^32^. Nevertheless, the magnitude of thinnest corneal thickness and central corneal thickness reduction was not statistically different between the treatment groups in this study, again suggesting a similar treatment efficacy in both groups.

We did not observe any inter-group difference in the improvement of UCVA, BCVA and spherical equivalent between conventional CXL and 18 mW/cm^2^ accelerated CXL in our study. Tomita *et al.* and Hashemian *et al.* showed similar visual outcomes between conventional CXL and 30 mW/cm^2^ accelerated CXL at one year^15^. These findings seem to suggest a similar clinical efficacy between the conventional and accelerated protocols, but long-term follow-up evaluation is warranted to delineate any difference in the clinical and topographical effect between the conventional and accelerated protocols. We did not observe visually significant corneal haze in any of our cases. This was despite using fluorometholone eye drops for a period of 3 weeks. We did not have to switch over to stronger topical corticosteroids. This may reflect a difference in treatment response in Chinese eyes. Further studies are necessary in a similar cohort to validate these observations.

The limitations of our study include a small sample size and lack of randomized study design. The sample size in this study can detect the observed change in maximum keratometry through repeated measures ANOVA test with power of 50%^33^. Measurement of difference in the depth of demarcation line would add credibility to the differences observed in the clinical effect of conventional CXL and accelerated CXL in our study. However, this has been demonstrated before^18^. We did not measure baseline and postoperative corneal dampening which has been variably shown to change after CXL^14^. We have endeavored to match both groups in terms of treatment protocols in order to make a fair comparison between conventional CXL and accelerated CXL. To conclude, we showed that both conventional CXL and 18 mW/cm^2^ accelerated CXL are effective in stabilizing progressive keratoconus at 1year. If there is a real difference between these two treatment modalities, we hypothesize that these effects should become evident over a long-term.

## Methods

### Study design

This was a prospective interventional study performed at the Department of Ophthalmology and Visual Sciences, Chinese University of Hong Kong. The Kowloon Ethics Committee approved the study protocol. The study was carried out in accordance with the approved protocol and the Declaration of Helsinki. All patients gave an informed consent before participation in the study. During the study period, we included consecutive patients with grade I–III (Amsler-Krumeich classification^34^) progressive keratoconus between 18 and 35 years of age. The diagnosis of keratoconus was based on corneal topography and its clinical signs such as stromal thinning, Fleischer ring, or Vogt striae. Patients with progressive keratoconus and deterioration in vision were advised to undergo CXL. The inclusion criteria for progression included loss of ≥2 lines of best-corrected visual acuity (BCVA) attributable just to keratoconus progression in addition to at least 1 of the following over the preceding 12 months: an increase of at least 1 diopter in the steepest keratometry value derived from computerized videokeratography, or, an increase in astigmatism determined by manifest subjective refraction of at least 1.0 D. Exclusion criteria included a minimum corneal thickness less than 400 μm, endothelial cell density less than 2000 cell/mm^2^, axial corneal scarring, previous corneal surgery, other corneal or ocular surface disorders, autoimmune diseases and pregnancy.

Conventional CXL was performed in 20 consecutive eyes between June 2011 and July 2012. Subsequently, we acquired another machine with an option to perform accelerated CXL, which was performed in 20 consecutive eyes between July 2012 and July 2013. All patients were Chinese. None of the patients were excluded during enrollment. Visual acuity was measured as Logarithm of the Minimum Angle of Resolution in this study. Patients in both groups had uncorrected visual acuity (UCVA), BCVA, slit-lamp examination (Slit Lamp BX900, Haag-Streit AG), corneal topography using scanning-slit imaging (Orbscan II, Bausch and Lomb Surgical, Salt Lake City, USA) and a semi-automated specular biomicroscopy utilizing the central method (Konan SP-9000, Hyogo, Japan) at baseline and 12 months after the treatment. Patients were requested to discontinue contact lens wear 2 weeks before evaluation or surgery during the study period. All images were acquired and analyzed in an unmasked manner.

### Treatment

All surgeries were performed in the same setting using a standard technique. Corneal anesthesia was achieved using proparacaine 0.5% ophthalmic solution. After surgical draping, a 9.0 mm corneal trephine was used to mark the central corneal treatment area. Epithelium was removed using a flat-edged blade knife. This was followed by application of 0.1% riboflavin with 20% dextran solution (MedioCROSS D, Kronen-Apotheke, Kiel, Germany) topically every 2 minutes for 30 minutes. Corneal thickness was measured using ultrasonic pachymetry (DGH 500, DGH Technology Inc., Exton, PA, USA).

Conventional CXL was performed using a 365-nm ultraviolet-A light (UV-X, IROC, Zurich, Switzerland) with 3-mW/cm^2^ irradiance for 30 minutes, while accelerated CXL was performed with a 365-nm ultraviolet-A lamp (CCL-Vario, Peschke Trade GmbH, Hünenberg, Switzerland) using 18-mW/cm^2^ irradiance for 5 minutes. The settings for both devices were similar in terms of working distance (5 cm), illumination diameter (9.0 mm), and, light emission (continuous wave). In all cases, riboflavin solution was reapplied every 2 minutes during the irradiation. A thin precorneal film of riboflavin was maintained during the irradiation. The corneal surface was thoroughly irrigated with balanced salt solution after irradiation. A bandage contact lens was placed over the cornea at the end of the procedure.

Postoperatively, all patients received oral analgesia in the form of acetaminophen 500 mg every 6 hours for the first 3 days, topical 0.5% levofloxacin eye drops four times daily for 1 week and preservative-free artificial tear drops four times a day for 1 month. Bandage contact lens was removed between 3–7 days postoperatively depending upon epithelial healing. After this, fluorometholone acetate 0.1% eye drops were commenced 4 times daily for 1 week and tapered off over a period of 3 weeks.

### Statistical analysis

Statistical analysis was performed using R 2.15.2 (R Foundation, Vienna, Austria). Baseline and 1-year postoperative parameters including maximum keratometry, minimum keratometry, thinnest corneal thickness, central corneal thickness, spherical equivalent, UCVA, BCVA and endothelial cell density were compared using one-way analysis of variance (ANOVA) models with repeated measures between conventional and accelerated corneal collagen crosslinking. The changes in maximum keratometry, minimum keratometry, thinnest corneal thickness, central corneal thickness, spherical equivalent, UCVA, BCVA and endothelial cell density were evaluated using one-way ANOVA with repeated measures for both groups. Two-way ANOVA with repeated measures was adopted to evaluate the change after CXL between both groups. Comparison of change in maximum keratometry and astigmatism 1-year before the surgery and pre-surgery in both groups was performed by two-way ANOVA with repeated measures. Association between the change in maximum keratometry, UCVA and BCVA with their baseline values was evaluated using linear mixed effect regression models to adjust for the correlation of fellow eyes for the two groups separately. Categorical variables between groups were compared using Fisher exact test. A p-value < 0.05 was considered statistically significant.

## Additional Information

**How to cite this article**: Chow, V. W. S. *et al.* One-year outcomes of conventional and accelerated collagen crosslinking in progressive keratoconus. *Sci. Rep.* **5**, 14425; doi: 10.1038/srep14425 (2015).

## Figures and Tables

**Table 1:**
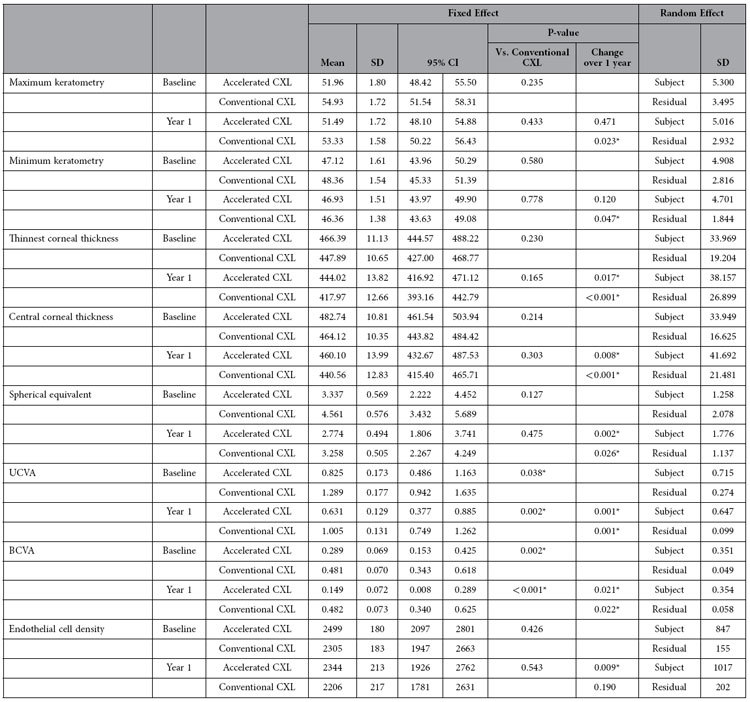
Comparison of 1-year outcomes between conventional and accelerated corneal collagen crosslinking in cases with progressive keratoconus.

CXL: crosslinking; SD: standard deviation; CI: confidence interval; UCVA: uncorrected visual acuity (LogMAR); BCVA: best-corrected visual acuity (LogMAR). * Statistically significant.

**Table 2:** Comparison of change in parameters over 1 year after conventional and accelerated corneal collagen crosslinking in cases with progressive keratoconus.

	**Change after 1 year**		
	**Conventional CXL**	**Accelerated CXL**	**p-value**
Maximum keratometry	−1.6 ± 0.72	−0.47 ± 0.83	0.343
Minimum keratometry	−2.00 ± 0.90	−0.19 ± 0.28	0.184
Thinnest corneal thickness	−29.91 ± 6.47	−22.38 ± 8.76	0.305
Central corneal thickness	−23.56 ± 5.56	−22.63 ± 7.98	0.606
UCVA	−0.28 ± 0.08	−0.20 ± 0.06	0.508
BCVA	0.00 ± 0.04	−0.14 ± 0.02	0.430
Spherical equivalent	−1.3 ± 0.53	−0.57 ± 0.26	0.554
Endothelial cell density	−99 ± 43	−105 ± 18	0.817

CXL: crosslinking; UCVA: uncorrected visual acuity (LogMAR); BCVA: best-corrected visual acuity (LogMAR).

**Table 3:**
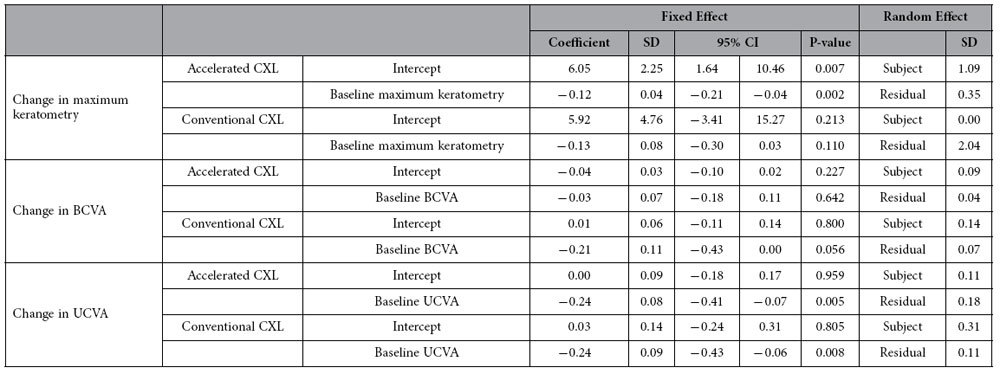
Association analysis between the baseline parameters based on linear mixed effect models after conventional and accelerated collagen crosslinking in cases with progressive keratoconus.

CXL: crosslinking; SD: standard deviation; CI: confidence interval; UCVA: uncorrected visual acuity (LogMAR); BCVA: best-corrected visual acuity (LogMAR).
